# CONCORDANCE IN SPOUSES’ INTENTION TO MOVE AFTER RETIREMENT AMONG KOREAN MIDDLE-AGED COUPLES

**DOI:** 10.1093/geroni/igad104.2634

**Published:** 2023-12-21

**Authors:** Eunju Lee, Kyungmin Kim

**Affiliations:** Seoul National University, Seoul, Republic of Korea; Seoul National University, Seoul, Republic of Korea

## Abstract

Midlife adults often envision life after retirement and start planning for retirement, forming their own expectations of where they live after retirement. Migration after retirement is an important decision in that physical and social environments of housing and communities affect health and well-being in later life. Research has emphasized that moving decisions are made at the household level, but less is known about whether middle-aged couples are concordant in their desire to move after retirement and whether concordant desires are associated with couples’ marital characteristics, such as marital satisfaction, dyadic coping styles, and communication. We analyzed a sample of 1,285 middle-aged couples (age 49–64) from the 2012 Korean Baby Boomer Panel Study and Korean Forgotten Generation Study. The majority of couples (83%) were concordant in their moving desires—by either agreeing to move (29%) or to stay (54%), whereas 17% of couples were not in agreement—either only husband desires to move (9%) or only wife desires to move (8%). Logistic/multinomial regression results showed that couples who had a communal problem-solving style and where the wife perceived higher marital satisfaction were more likely to be concordant in their moving desires (i.e., agreeing to move or to stay), after controlling for other individual and family characteristics. Further, couples were more likely to agree to move—when they had discussion about life after retirement and shared individual stress between spouses. Our findings suggest the importance of marital dynamics in understanding concordance in spouses’ desire to move after retirement.

